# Epithelioid Sarcoma Presenting as Non-Healing Traumatic Ulcer: A Case Report and Review of the Literature

**DOI:** 10.7759/cureus.14014

**Published:** 2021-03-20

**Authors:** Shiyas Mohammedali, Sohail J Quazi, Mohammed Muneer, Mazin Mohammed, Atalla Hammouda

**Affiliations:** 1 Plastic Surgery, Hamad Medical Corporation, Doha, QAT; 2 Plastic and Reconstructive Surgery, Hamad Medical Corporation, Doha, QAT; 3 Plastic Surgery, California Institute of Behavioral Neurosciences & Psychology, Fairfield, USA

**Keywords:** epithelioid sarcoma, chronic ulcer, traumatic ulcer, atypical presentation of epithelioid sarcoma

## Abstract

Epithelioid sarcoma is a rare soft tissue sarcoma. It is a slow-growing neoplasm, which usually presents as a painless mass in the extremities and typically grows along deep dermal and subcutaneous planes. In contrast to other types of sarcoma, it has a strong tendency for nodal metastasis and local metastasis adjacent to the primary lesion within the affected limb.

In this article, we present a case of chronic traumatic ulcer in the upper extremity in an adolescent male that was subsequently diagnosed as epithelioid sarcoma, which is a very unusual mode of presentation of this particular tumour. The patient was treated with wide local excision and reconstruction with a free flap. Histopathological examination and immunochemistry studies confirmed the diagnosis and the patient underwent radiotherapy post-operatively as a part of the treatment regime. His post-treatment period was unremarkable, and he was put on regular surveillance to monitor the development of any signs of disease recurrence. Patients with epithelioid sarcomas often present late due to the slow-growing nature of the tumour. Unusual presentations like this will further delay the diagnosis and treatment, which will eventually worsen the prognosis. Awareness of such presentations can encourage primary care physicians to make early referrals to experts, which, in turn, may help the patients get early treatment and have a better prognosis.

## Introduction

Epithelioid sarcoma, a rare neoplasm that makes up less than 1% of all soft tissue sarcomas, is a slow-growing neoplasm seen mainly in the adolescent and young population. First described by Enzinger in 1970, it typically presents as a deep dermal or subcutaneous mass in the distal aspect of the upper extremity. Though a slow-growing neoplasm, it has a strong predisposition for spreading to regional lymph nodes and local recurrence in the vicinity of the primary tumour, in contrast to other types of sarcoma [1]. Epithelioid sarcoma is seen rarely in children and the elderly, but young people seem to be most affected, with the highest incidence reported among people between 20 and 30 years old.

Due to its slow-growing nature, epithelioid sarcoma often tends to be misdiagnosed as a benign lesion, thus delaying the diagnosis and treatment. The time between detecting the first symptom and commencement of treatment ranges between one and 36 months, with the median being 3.5 months [2]. This sarcoma has a relatively high rate of recurrence and distant metastasis (77% and 45%, respectively). The lung constitutes 51% case of metastatic cases. The next most common site is regional lymph nodes (34%), followed by the scalp (22%) and bone (13%) [3]. Epithelioid sarcoma has five- and 10-year survival rates of 50-70% and 42-55%, respectively [4]. Several factors are implicated in the prognosis of the disease, such as gender, site, age at diagnosis, tumour size and histological findings [4,5]. Worst outcomes are often associated with male gender, proximal rather than distal lesions [5], late age at presentation, tumours larger than 2 cm across, vascular invasion, necrosis and high mitotic index [6].

## Case presentation

**Figure 1:**
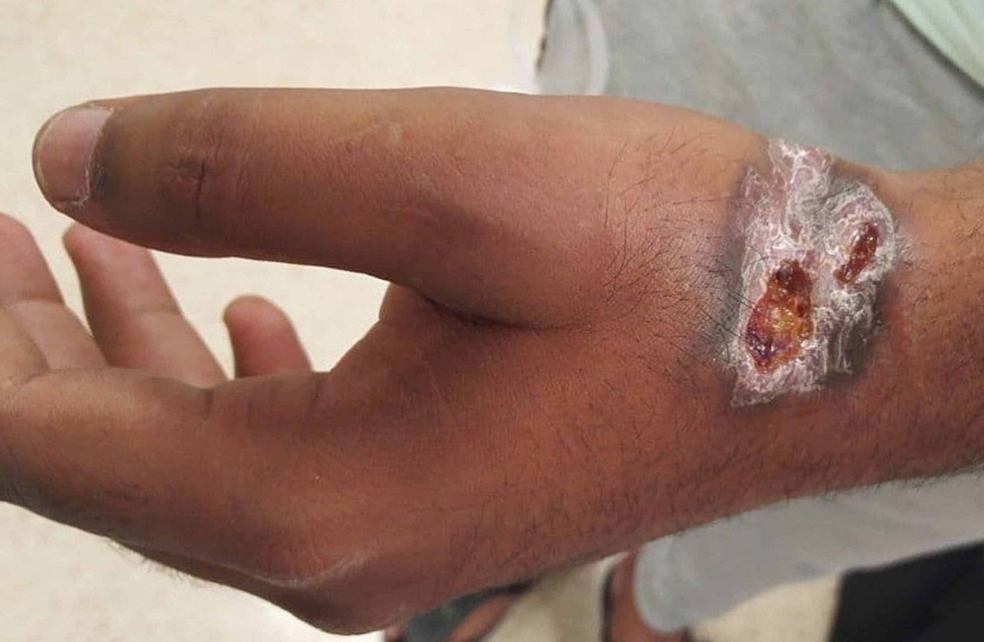
Chronic non-healing ulcer over left wrist Elliptical wound of 6x4 cm, with areas of scarring and ulceration in the floor, keratotic edges and hyper-pigmented margins.

A 21-year-old male, not known to have any chronic illness, was seen in the outpatient department. He had sustained a superficial laceration to the left wrist in a road traffic accident 18 months previously and had been undergoing regular wound care at a peripheral health centre since. There was no documented history of any surgical procedure or episode of wound infection. Despite the management, the wound had not healed completely; rather it healed partially with eschar and unhealthy granulation tissues. Surrounding skin became thin and hyperpigmented. Examination revealed a well-defined elliptical ulcer with irregular edges, 6x4 cm in size, over the radial border of the wrist. There were areas of scarring within the ulcer and on the surrounding skin. The floor had scattered pale granulations, and the edges were keratotic (Figure 1). The wound looked deep, affecting the underlying structures, including the tendons and joint. Thumb flexion, extension and abduction were limited due to pain and joint stiffness.

**Figure 2:**
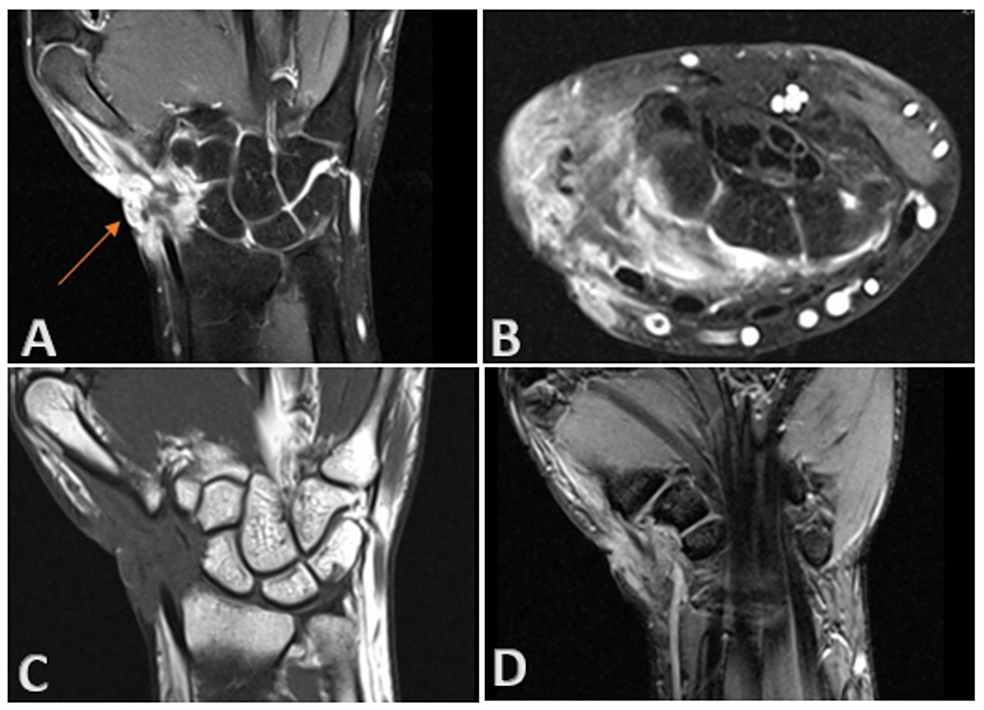
Epithelioid sarcoma: MRI with contrast. MRI wrist showing peripherally enhancing lesion within the soft tissues of the lateral aspect of the wrist joint, communicating with the cutaneous defect (A). Adjacent soft tissues show extensive inflammatory changes extending to the intercarpal region and dorsal aspect of wrist joint (B,C,D). Adjacent synovium also shows diffuse thickening and enhancement (B). Bony components show normal marrow signal intensity without evidence of edema. (A, C and D are coronal sections; B is cross section)

**Figure 3:**
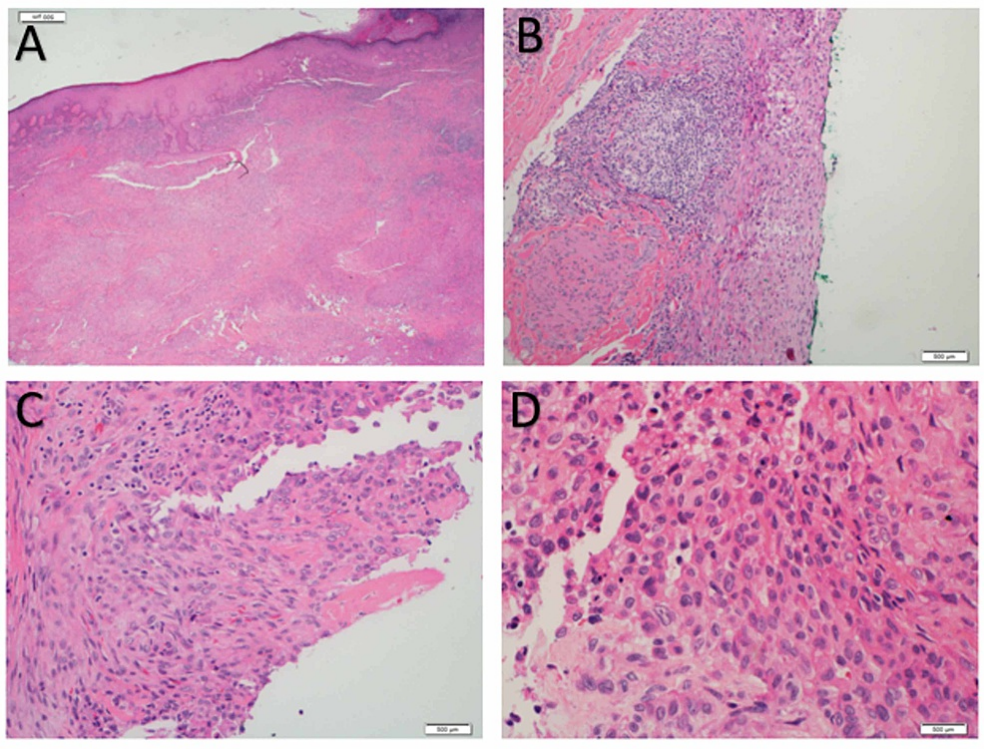
Epithelioid sarcoma: histopathology. Sections show subcutaneous proliferation of nodules and sheets of epithelioid cells with large vesicular eccentric nuclei and nucleoli with abundant eosinophilic cytoplasm, some of which appear as pseudo-granulomas surrounding central necrosis. Focally, the epithelioid cells merge into spindle cells embedded in desmoplastic fibrous stroma with focal atypical nuclear features. Also, bland spindle cells in a storiform pattern can be seen (A and B are low power fields; C and D are high power fields )

**Figure 4:**
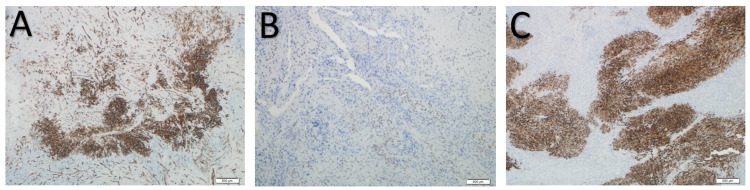
Epithelioid sarcoma: immunohistochemistry. Immunohistochemistry is diffusely positive for vimentin, epithelial membrane antigen (EMA) and cytokeratin 8; focally positive for smooth muscle actin, CD34, cytokeratin 19 and integrase interactor 1 (INI-1 [A-CD34, B-IN1 and C-EMA]); and negative for myogenin, p63, desmin, cytokeratin 7, cytokeratin 5/6, cytokeratin 14, CD 117, Melan-A, HMB-45, S100, Factor 13A and CD68.

Since the duration and wound features were suspicious of a malignant lesion, an MRI scan was ordered. It showed a peripherally enhancing soft tissue lesion with inflammatory changes, communicating with an ulcer on the lateral aspect of the wrist (Figure 2). Adjacent soft tissue showed extensive inflammatory changes extending to the intercarpal area and dorsal aspect of the wrist joint. Synovium also showed diffuse thickening and enhancement. The patient underwent excision biopsy of the lesion with a 2-cm margin. The lesion was found to be ill-defined intra-operatively, growing along the fascial planes, and invading the dorsal capsule of the first and second carpometacarpal joints. The lesion was also seen affecting the paratenon of the first, second and third extensor compartment tendons. Complete excision of all affected structures, including the affected joint capsules and paratenon was performed, leaving the tendon substance intact. Histopathological examination of the lesion was confirmatory for epithelioid sarcoma, with positive, deep margins (Figure 3). The immuno-histochemistry was diffusely positive for epithelial membrane antigen (EMA), cytokeratin 8 and vimentin; it was focally positive for cytokeratin 19, smooth muscle actin, CD34 and integrase interactor 1 (INI-1); and it was negative for myogenin, p63, desmin, cytokeratin 7, cytokeratin 5/6, cytokeratin 14, CD117, Melan-A, HMB-45, S100, Factor 13A and CD68 (Figure 4).

**Figure 5:**
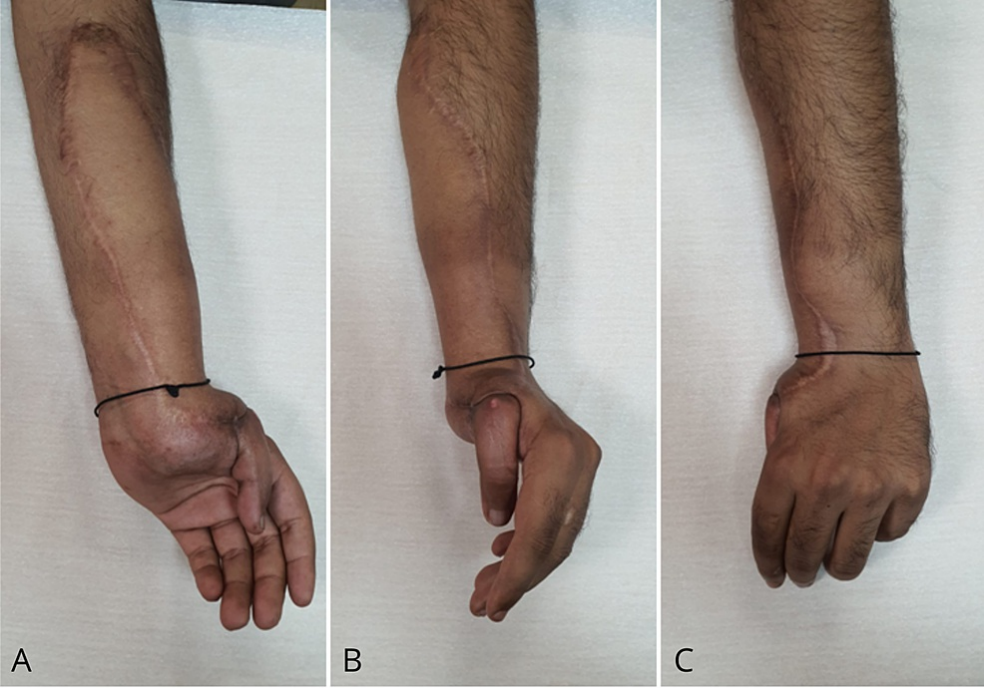
Epithelioid sarcoma: clinical picture at four year follow up Left wrist and distal forearm showing well settled flap with good contour and inconspicuous scars. (A is volar, B is radial and C is dorsal view)

The case was referred to the multidisciplinary team to plan further treatment. As per their recommendation, the patient underwent re-excision of the lesion with wider margins, and the resultant defect was covered with an antero-lateral thigh free flap. He received adjuvant radiotherapy with 50 Grays in 25 daily fractions plus 10 Grays in five daily fractions booster. Further investigations, including sentinel lymph node biopsy and positron emission tomography (PET) scan, revealed no local or distant metastasis. He was followed up for four years in outpatient clinic regularly and MRI scan was performed every six months. Clinical examination during the latest visit revealed a well-settled flap with inconspicuous scars (Figure 5). He did not develop any signs of disease recurrence or distant spread throughout the follow-up period. He had persistent pain, stiffness and limited range of motion in the affected wrist and fingers, for which he is currently undergoing treatment under physiotherapy and orthopaedic departments.

## Discussion

Epithelioid sarcoma is a highly malignant tumour known for its tendency to recur locally and metastasize to lymph nodes and distant organs [1]. Fifty-four percent of cases occur in the hand and forearm, followed by the lower limb and the proximal upper limb. Epithelioid sarcoma is relatively uncommon, accounting for less than 1% of all sarcomas affecting soft tissue [3]. Most lesions are found either in the dermis or deep soft tissue as firm-to-hard palpable masses. Superficial lesions may ulcerate and appear like a wart or chronic non-healing ulcer, while deep-seated tumours sometimes resemble ganglion cysts or giant cell tumours if attached to tendons. Pain or tenderness is rare, affecting only 20% of patients when large nerves are affected by the tumour [3]. Long-standing hand tumours may present with joint or soft tissue contractures or symptoms of nerve compression, like numbness or muscle weakness. Epithelioid sarcoma is highly malignant; 32% of people affected die from the disease. Unlike other soft tissue sarcomas, epithelioid sarcomas have a strong predisposition for regional nodal metastasis [6]. In a long-term study, metastatic disease was found in 45% of cases of epithelioid sarcoma, with the lung being the most common site, followed by regional lymph nodes and the scalp.

MRI is the investigation of choice for diagnostic imaging, which helps primarily in defining the anatomic extent of the tumour and also differentiating recurrence from postoperative changes. As in other soft tissue sarcomas, the diagnostic confirmation is tissue biopsy. The pathologic features of epithelioid sarcoma are often inconsistent, so interpretations of the microscopic specimen and biopsy should be performed by a pathologist and experienced musculoskeletal oncologist, respectively. Microscopically, these tumours show nodules of epithelioid and spindle cells, forming a granuloma-like pattern with areas of necrosis and central hyalinization. Some less common histologic variants have been reported, such as angiomatoid- and fibrous histiocytoma-like sub-types. In epithelioid sarcoma, the cellular elements consist of deep eosinophilic cytoplasm and vary in shape from (large) ovoid through polygonal to spindle. The most important tool in making the diagnosis of epithelioid sarcoma is immuno-histochemical examination [7]. The staining pattern indicates that the origin of epithelioid sarcoma is mesenchymal with epithelial dedifferentiation. Epithelioid sarcoma is characterized by expression of low-molecular-weight cytokeratins and vimentins, especially those identified by the monoclonal antibody cell adhesion molecule 5.2 [8,9], and a majority (85-96%) of cases are epithelial membrane antigen-positive [10].

The treatment of choice of epithelioid sarcoma is wide surgical resection. According to some studies, resection of the tumour should be radical, despite the morbidity it may lead to, as the recurrence rates after marginal resection are up to 77% [11]. However, some authors recommend less radical treatments for local control as epithelioid sarcoma can metastasize locally in the same limb, proximally to the primary tumour. In areas like fingertips, where organ loss causes minimal morbidity, amputation can be considered if tumour recurs multiple times. Surgical treatment alone can be an option in selected patients with primary soft tissue sarcomas of the extremity when there are acceptable local disease control and good long-term survival [12]. Some authors recommend sentinel lymph node sampling as epithelioid sarcoma has a predisposition for metastasis to regional lymph nodes. Although chemotherapy (doxorubicin) has not been shown to improve survival, it has been used in certain cases, including metastatic and multifocal diseases and tumours larger than 5 cm. The late effects of radiotherapy, such as scarring, stiffness and neuropathy, can lead to increased disability of the hand.

Literature review

In 2016, Nishibaba et al. reported a case of epithelioid sarcoma in a 74-year-old woman that was misdiagnosed as a fungal infection. Failure of initial treatment prompted a re-biopsy of the lesion, which surprisingly revealed lymphocyte infiltration and proliferating atypical oval or polygonal epithelioid cells along the dermis. Immunohistochemistry tests were positive for cell adhesion molecule 5.2, E26-related gene, vimentin and epithelial membrane antigen. They concluded with a recommendation for repeat biopsy and immuno-histochemical studies in cases that are resistant to conventional treatment methods, with epithelioid sarcoma included as one of the differential diagnoses [13]. Guillou et al. reported a case of proximal-type epithelioid sarcoma in 1997. This is found mainly in the genital tracts and pelvic region of middle-aged and young adults. Histo-pathologically, it does not show the typical granuloma-like pattern but rather a characteristic proliferation of epithelioid-like cells with rhabdoid features [14]. In 2016, Echchaoui et al. reported a case of progressively enlarging nodule in the scapular region that was initially excised but presented again with recurrent growth. A wider excision was performed with a 4-cm margin. Histopathology and immunohistochemistry studies confirmed the diagnosis. Surgical resection was the only treatment, and the patient did not develop any recurrence of the lesion at 18 months of follow-up [15].

In 2013, Mendes dos Santos et al. reported a case of proximal-type epithelioid sarcoma in a 25-year-old man. This presented as a rapidly progressing and painful lesion over his right buttock of three months duration and eventually ulcerated. Diagnosis of proximal type-epithelioid sarcoma was made from immuno-histochemical and histopathological studies. A staging CT scan revealed multiple metastases in the lungs and brain. The tumour exhibited an aggressive clinical course, with rapid distant metastasis and death nine months after diagnosis [16]. Stang et al. reported a case of an 11-year-old girl who presented with a month-old swelling over the distal phalanx of the left middle finger. Initial management was performed by a non-hand surgeon, who suspected ganglion and excised it after seeing a normal preoperative radiograph. A specimen was sent for histopathological examination, which showed epithelioid sarcoma with an incompletely excised tumour. Hence, a wider resection was performed in the tumour bed. No residual tumour was found in a repeat histopathological examination, so the resultant defect was closed with a split-thickness skin graft. The patient did not receive further chemotherapy and radiotherapy, and almost 1.5 years later, when the case report was made, there was no evidence of recurrence [17].

## Conclusions

Epithelioid sarcoma is a rare and aggressive neoplasm with high local recurrence rates and early nodal and distant metastases. For early detection and treatment, it should be considered one of the differential diagnoses when encountering a young adult with an unusual presentation of traumatic wounds and non-healing ulcers. An MRI scan is the initial diagnostic investigation of choice, and biopsy is necessary for confirmation. Deeper biopsies are recommended to demonstrate the overtly malignant morphological characteristics of epithelioid sarcoma. Once the diagnosis is confirmed, prompt treatment should be launched as any delay will worsen the prognosis. Common treatment modalities include wide surgical excision and radiotherapy. Postoperative periodic surveillance with MRI scan is the key to early detection of disease recurrence.
